# The impact of artificial selection for *Wolbachia*-mediated dengue virus blocking on phage WO

**DOI:** 10.1371/journal.pntd.0009637

**Published:** 2021-07-27

**Authors:** Heverton L. C. Dutra, Suzanne A. Ford, Scott L. Allen, Sarah R. Bordenstein, Stephen F. Chenoweth, Seth R. Bordenstein, Elizabeth A. McGraw

**Affiliations:** 1 Department of Biology, Pennsylvania State University, University Park, Pennsylvania, United States of America; 2 Center for Infectious Disease Dynamics, Huck Institutes of the Life Sciences, University Park, Pennsylvania, United States of America; 3 School of Biological Sciences, The University of Queensland, St. Lucia, Queensland, Australia; 4 Department of Biological Sciences, Vanderbilt University, Nashville, Tennessee, United States of America; 5 Vanderbilt Microbiome Initiative, Vanderbilt University, Nashville, Tennessee, United States of America; 6 Department of Pathology, Microbiology, and Immunology, Vanderbilt University, Nashville, Tennessee, United States of America; 7 Vanderbilt Institute for Infection, Immunology and Inflammation, Vanderbilt University Medical Center, Nashville, Tennessee, United States of America; University of Maryland Baltimore, UNITED STATES

## Abstract

*Wolbachia* is currently at the forefront of global efforts to control arbovirus transmission from the vector *Aedes aegypti*. The use of *Wolbachia* relies on two phenotypes—cytoplasmic incompatibility (CI), conferred by *cifA* and *cifB* genes in prophage WO, and *Wolbachia*-mediated pathogen blocking (WMPB). These traits allow for local, self-sustaining reductions in transmission of dengue (DENV) following release of *Wolbachia*-infected *A*. *aegypti*. Here, aided by previous artificial selection experiment that generated Low and High pathogen blocking lines, we examined the potential link between WMPB and phage WO. We found no evidence that *Wolbachia* or phage WO relative densities predict DENV blocking strength across selected lines. However, selection resulted in reduced phage WO relative density for the Low WMPB line. The Low blocking line was previously shown to have reduced fitness as a result of selection. Through subsequent genomic analyses, we demonstrate that SNP variation underpinning selection for low blocking led to elevated frequency of potential deleterious SNPs on chromosome 1. The key region on chromosome 1 contains genes relating to cell cycle regulation, oxidative stress, transcriptional pausing, among others, that may have cascading effects on *Wolbachia* intracellular environment. We hypothesize that reduction in phage WO may be driven by changes in the loci directly under selection for blocking, or by the accumulation of predicted deleterious alleles in linkage disequilibrium with blocking loci resulting from hitchhiking. For the Low line with fewer phage WO, we also detected reduced expression of *cifA* and *cifB* CI genes, with patterns of expression varying between somatic and reproductive tissues. In conclusion, we propose that artificial selection for WMPB trait had corresponding impacts on phage WO densities, and also the transcription of CI-causing genes. Future studies may include a more detailed analysis of the regions the *A*. *aegypti* chromosome 1’s ability to affect WMPB and other *Wolbachia*-associated intrinsic factors such as phage WO.

## Author summary

*Wolbachia* are widespread endosymbiotic bacteria of insects that cause *Wolbachia*-mediated pathogen blocking (WMPB) and cytoplasmic incompatibility (CI). The latter mediated by *cif* genes localized in the prophage WO region. Because of that, *Wolbachia*-infected mosquitoes are currently being used in field to fight the transmission of vector-borne viruses such as Dengue (DENV) to human populations. Aided by a previous artificial selection experiment that generated lines with variable (High and Low) DENV blocking strength, we tested for a potential link between WMPB and phage WO. There was no evidence that *Wolbachia* nor phage WO densities predict DENV blocking strength. However, we found that the Low blocking line had reduced phage WO density, and lower expression of the *cif* genes in a tissue-specific manner. We demonstrate that in addition to previous report of reduced fitness, the Low blocking line also exhibited increased frequency of potential deleterious SNPs on chromosome 1. Our hypotheses are that reduction in phage WO may have resulted from changes in the loci directly under selection for blocking, or by linkage disequilibrium events linked to the accumulation of mosquito predicted deleterious alleles. Our results highlight the importance of chromosome 1 for WMPB and its potential impact for other *Wolbachia*-associated factors like phage WO.

## Introduction

Mosquitoes transmit many medically relevant pathogens [1]. *Aedes aegypti* is a relevant vector of arboviruses as it transmits dengue (DENV), Zika, chikungunya and yellow fever viruses. DENV infection causes approximately 100 million symptomatic infections per year [2], with an estimated global annual cost to health care systems of US $8–9 billion [3]. Currently, half the world’s population is at risk of dengue infection. The risk will likely intensify, as the vector’s geographical range is predicted to increase due to climate change and socioeconomic factors [2,4]. Without an effective antiviral drug or vaccine [5], vector control remains the best strategy to curb vector-borne disease transmission.

Classical vector control initiatives such as active removal of mosquito breeding sites and insecticide spraying have limitations such as poor community compliance and development of insecticide resistance [6–8]. As such, the development of alternative approaches like gene drives [9,10] and the use of bacteria and fungi for biocontrol [11] is critical. Amongst these, the use of *Wolbachia* is one of the most promising biological strategies for controlling arbovirus transmission in the field [12,13]. *Wolbachia* is a genus of maternally-inherited, endosymbiotic bacteria present in many arthropod species [14]. The bacteria can induce two main effects in mosquitoes that together make them useful for biological control. First, they reduce the replication of co-infecting arboviruses, a phenotype known as “*Wolbachia*-mediated pathogen blocking” (WMPB) [15,16]. Second, they manipulate host reproduction in a manner that enhances the symbiont’s spread in arthropod populations through induction of a phenotype known as cytoplasmic incompatibility (CI) [17]. CI is typically characterized by reduced viability of embryos from matings specifically between *Wolbachia*-free females and *Wolbachia*-infected males [17,18]. *Wolbachia*-infected females, in contrast, reproduce successfully regardless of their mate’s infection status and produce more infected offspring; hence, *Wolbachia* spreads. Numerous field releases of *A*. *aegypti* stably transinfected with *Wolbachia*, have demonstrated the symbiont’s ability to spread, and more recently, to reduce the transmission of DENV to humans [12,13,19].

The genetic basis of *w*Mel-induced CI was recently resolved to two genes, here referred as *cytoplasmic incompatibility factor A* (*cifA*) *and cytoplasmic incompatibility factor B* (*cifB*), in the eukaryotic association module of phage WO (named after *Wo**lbachia*) [20–22] (please refer to [23–25] for an in-depth discussion on the proposed nomenclature of CI genes according to phylogenetic and enzymatic data on the matter). Considering all insect hosts surveyed, phage WO is temperate and may exist as a resident in *Wolbachia* genomes containing prophage WO (integrated lysogenic form in *Wolbachia* chromosome) or as a lytic bacteriophage (infectious capsids capable of exiting *Wolbachia*) [26–28]. Prophage WO and/or its genetic remnants are present in at least 89% of surveyed *Wolbachia* strains [29]. Most *Wolbachia* genomes are known to contain multiple phage WO haplotypes, or genetic variants of the phage WO family. In most cases, defective forms of the prophage WO (unable to form a lytic particle) are generally accompanied by fully-intact phage [22,30]. For instance, the genome of *w*Mel contains two prophage elements: a small pyocin-like element named WOMelA and a larger intact prophage named WOMelB. It is still unknown if WOMelB is capable of forming active lytic particles, and without complete reference genomes for both the integrated prophage and active phage, there is no way of precisely distinguishing between lysogenic and lytic particles. WOMelB modular structure is intact, however, and composed of two prophage fragments that complement each other, WOMelB1 (containing the head and tail modules), and WOMelB2 (harboring the baseplate module and recombinase) [30–32].

Although phage WO commonly exists in multiple copies [31,33] and comprises a significant percentage of *Wolbachia’s* genome, only a single pair of *cifA* and *cifB* is required to recapitulate CI in transgenic *D*. *melanogaster* [21,34]. Furthermore, rescue of CI can occur by expression of *cifA* alone in ovaries [20,35]. While the expression of CI assists *Wolbachia* spread, WO is not entirely beneficial as described in the “phage density model”. When in the lytic phase, mature phage WO particles lyse *Wolbachia*, leading to corresponding decreases in bacterial density and consequentially CI expression because the *cif* genes reside in the prophage region of *Wolbachia*.

The mechanism of WMPB in mosquitoes remains unsolved. Several factors are associated with WMPB [36–39]. Recently, we employed experimental evolution in an attempt to solve the mechanism of WMPB, as well as to predict its likely long-term evolutionary stability [40]. We artificially selected for both stronger (referred throughout the manuscript as High) and weaker (referred throughout the manuscript as Low) DENV blocking in mosquitoes infected with *w*Mel *Wolbachia*. In the resulting selected lines, our group tested for differences in mosquito fitness between evolved populations, and the associated differences in SNPs in the mosquito genome [40,41]. The study revealed two novel major gene candidates, α-mannosidase, and cadherin, along with another 58 minor genes in *A*. *aegypti* whose SNP variation associated with changes in WMPB phenotype. It also revealed that mosquito lines selected for higher blocking also exhibited faster population growth rates, an association that may be due to pleiotropy between the two traits. This association may help natural selection maintain or drive strong blocking in populations.

While the mechanistic basis of both CI and pathogen blocking are two ‘holy grails’ of *Wolbachia* biology, it has not been explored whether there may be interactions between these two traits. Here, we took advantage of the above artificial selection experiment, where we generated lines exhibiting phenotypic extremes in blocking. Using DNA and RNA samples from these lines, we tested for correlations between blocking strength and SNP variation, and the loads of both *Wolbachia* and phage WO. Last, we examined *cifA* and *cifB* gene expression in tissues of the selected lines to explore the potential consequences of selection on blocking for the expression of genes known to impact the strength of CI [20].

## Results

### Artificial selection on DENV blocking strength alters phage-*Wolbachia* associations

We quantified the parameters related to both *Wolbachia* and phage WO densities utilizing a set of samples previously collected as part of the original selection experiment by Ford *et al*. [40]. We provide this quantification for samples collected from two lines (each in triplicate) post four generations of selection for High and Low DENV blocking as per Ford *et al*. [40]. In this study, both the Ancestral base population and the Random selected lines were excluded from our analyses since neither of the comparisons act as true controls to the evolved lines (see Materials and methods sub-section “mosquitoes” for details).

**Fig 1:**
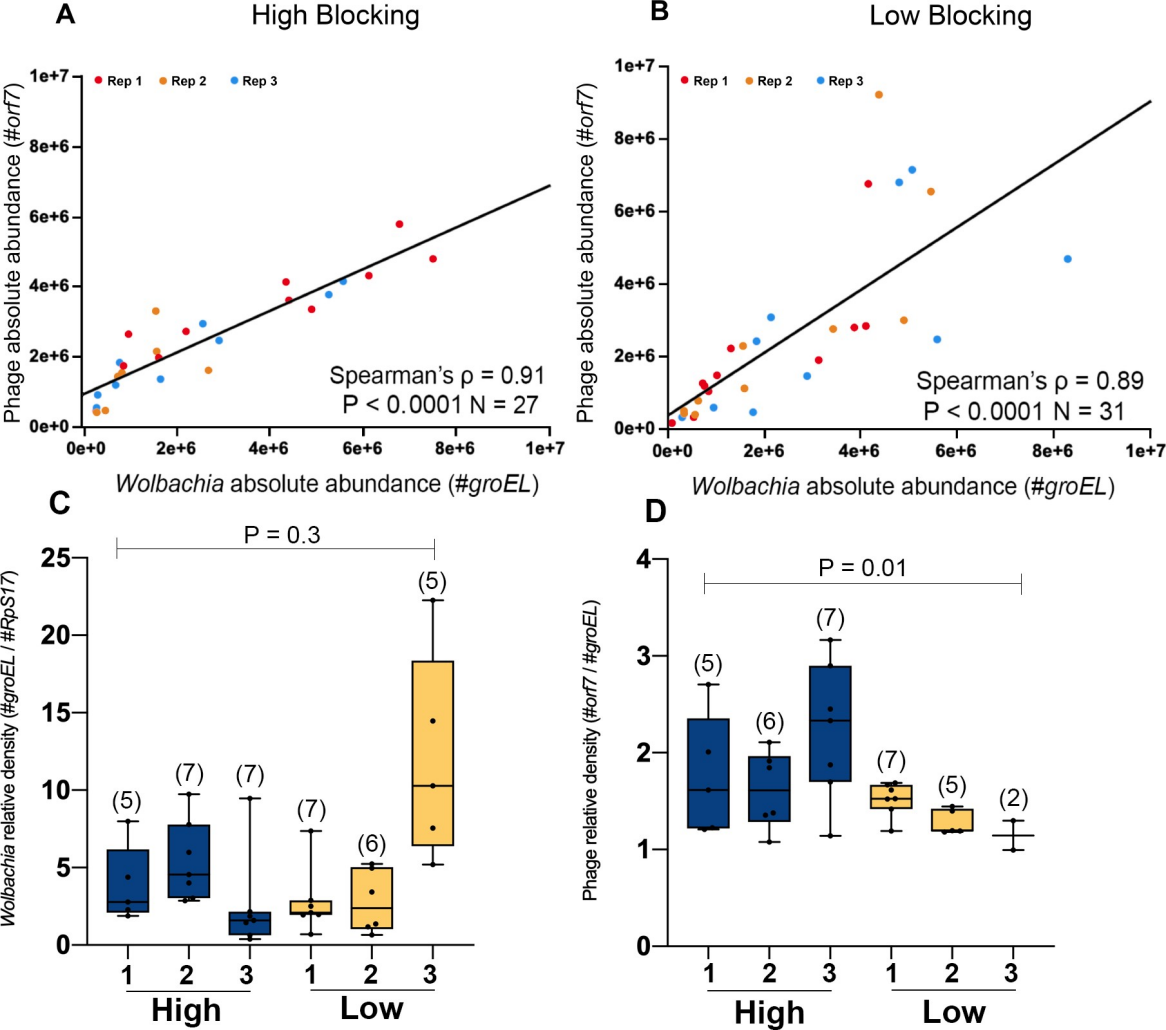
Artificial selection for variable *Wolbachia*-mediated DENV blocking strength causes alteration in phage WO relative density, with *Wolbachia* and phage WO absolute abundances positively correlating in *A*. *aegypti*. Whole body (minus ovaries) *Wolbachia* and phage relative densities for *w*Mel-infected *A*. *aegypti* mosquitoes from the evolved lines after 4 generations of selection (High: dark blue—Low: yellow). Correlation between *Wolbachia* (absolute copy number of *groEL*) and phage WO (absolute copy number of *orf7*) abundances in whole body (minus ovaries) of *w*Mel-infected *A*. *aegypti* mosquitoes selected for (A) High and (B) Low WMPB. Red, orange and blue dots represent replicated populations 1, 2 and 3, respectively. (C) *Wolbachia* densities are reported as density per host cell (absolute copy number of *groEL* relative to the absolute copy number of *rps17*) and (D) phage density as per *Wolbachia* cell (absolute copy number of *orf7* relative to the absolute copy number of *groEL*). Each dot represents a single mosquito and Boxplot depict medians. Sample sizes for each group are depicted on each graph. Numbers “1”, “2” and “3” represents distinct replicated populations of the same selected group. Significant comparisons expressed via P-values were determined via Spearman’s rank correlation coefficient across evolved groups for graphs (A) and (B). Graphs (C) and (D) were analyzed via Mann-Whitney U test.

We first examined the absolute abundances of both *Wolbachia* and phage WO in the evolved lines (Fig 1A and 1B) (High and Low blocking) and saw a strong positive correlation between the two variables, as expected. Next, we utilized a relative density measurement to explore the interaction between phage WO and *Wolbachia* (see materials and methods sub-section “*Wolbachia*, phage and mosquito gene quantification” for details on quantification strategies). Relative phage WO densities can estimate lytic phage development [26] when the number of phage genomes relative to *Wolbachia* genomes exceeds the baseline expectation of one prophage WO copy for every *w*Mel genome. Alternatively, molecular makers for phage WO genome rearrangements can be used to distinguish lytic versus lysogenic genomes [42], but that is not currently possible as the complete genome sequence for WOMelB phage particles has not yet been resolved. Whole-body analyses (minus ovaries) on individuals revealed that while *Wolbachia* relative density values were similar between the two evolved populations (Mann-Whitney U = 364, d.f. = 1; P = 0.3) (Fig 1C), phage WO relative densities were significantly lower in the Low blocking group (1.38 ± 0.21 –mean ± standard error) compared to High blocking (1.89 ± 0.62) (Mann-Whitney U = 63, d.f. = 1; P = 0.01) (Fig 1D). This finding indicates alterations on phage relative density between the evolved populations as a consequence of selection for WMPB.

**Fig 2:**
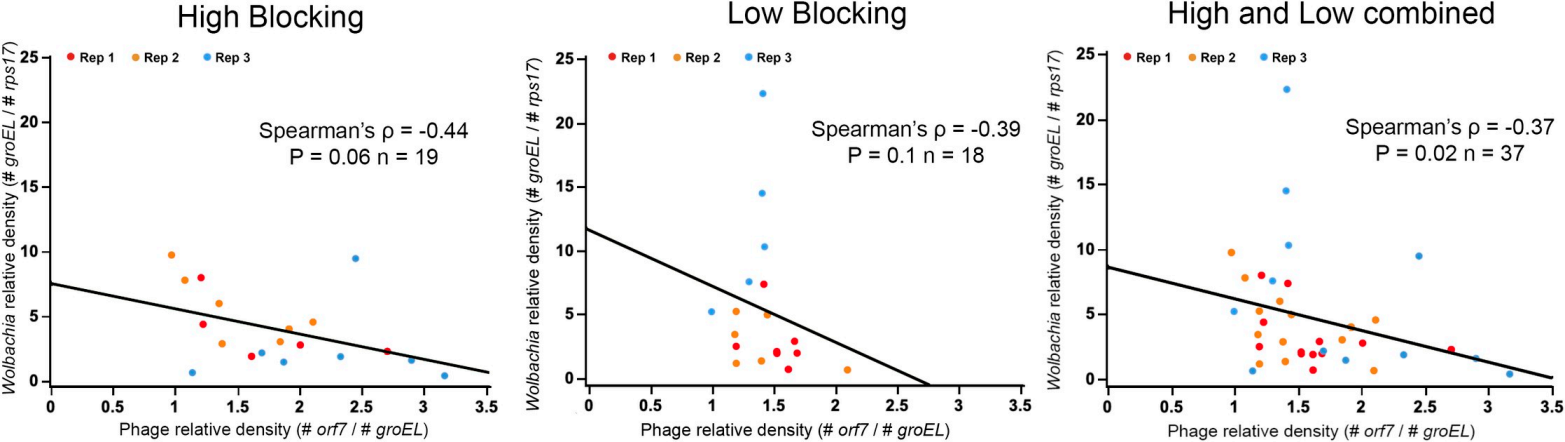
*Wolbachia* relative density inversely associates with phage WO relative density in evolved populations of *A*. *aegypti* regardless of selection. Correlation between *Wolbachia* and phage WO relative densities in whole body (minus ovaries) *w*Mel-infected *A*. *aegypti* mosquitoes selected for High and Low WMPB. Each dot represents a single mosquito. Sample sizes are depicted within the figure for each group. Red, orange and blue dots represent replicated populations 1, 2 and 3, respectively. Spearman’s rank correlation coefficient analyses were performed to describe the relationship between *Wolbachia* density (absolute copy number of *groEL* relative to the absolute copy number of *rps17*) and phage WO density (absolute copy number of *orf7* relative to the absolute copy number of *groEL*).

We then tested for correlations between the relative densities of both *Wolbachia* and phage WO in selected populations using Spearman’s correlation coefficient analysis, as to test for the phage density model [26]. We observed marginally non-significant, negative correlations for both the High (Spearman’s ρ = -0.44, P = 0.06) and Low blocking groups (Spearman’s ρ = -0.39, P = 0.12). The significance values of these correlations are very likely due to sample sizes, as combining both sets of selected lines resulted in a significant and expected negative correlation of relative phage WO density against *Wolbachia* density (Spearman’s ρ = -0.37, P = 0.02) (Fig 2). Under the phage WO density model, high *Wolbachia* densities associate with baseline prophage WO densities of about 1 in this case, and low *Wolbachia* densities associate with higher phage WO densities likely due to phage WO replication and lysis of *Wolbachia* cells.

### Neither phage nor *Wolbachia* density predicts the overall DENV blocking phenotype

**Fig 3:**
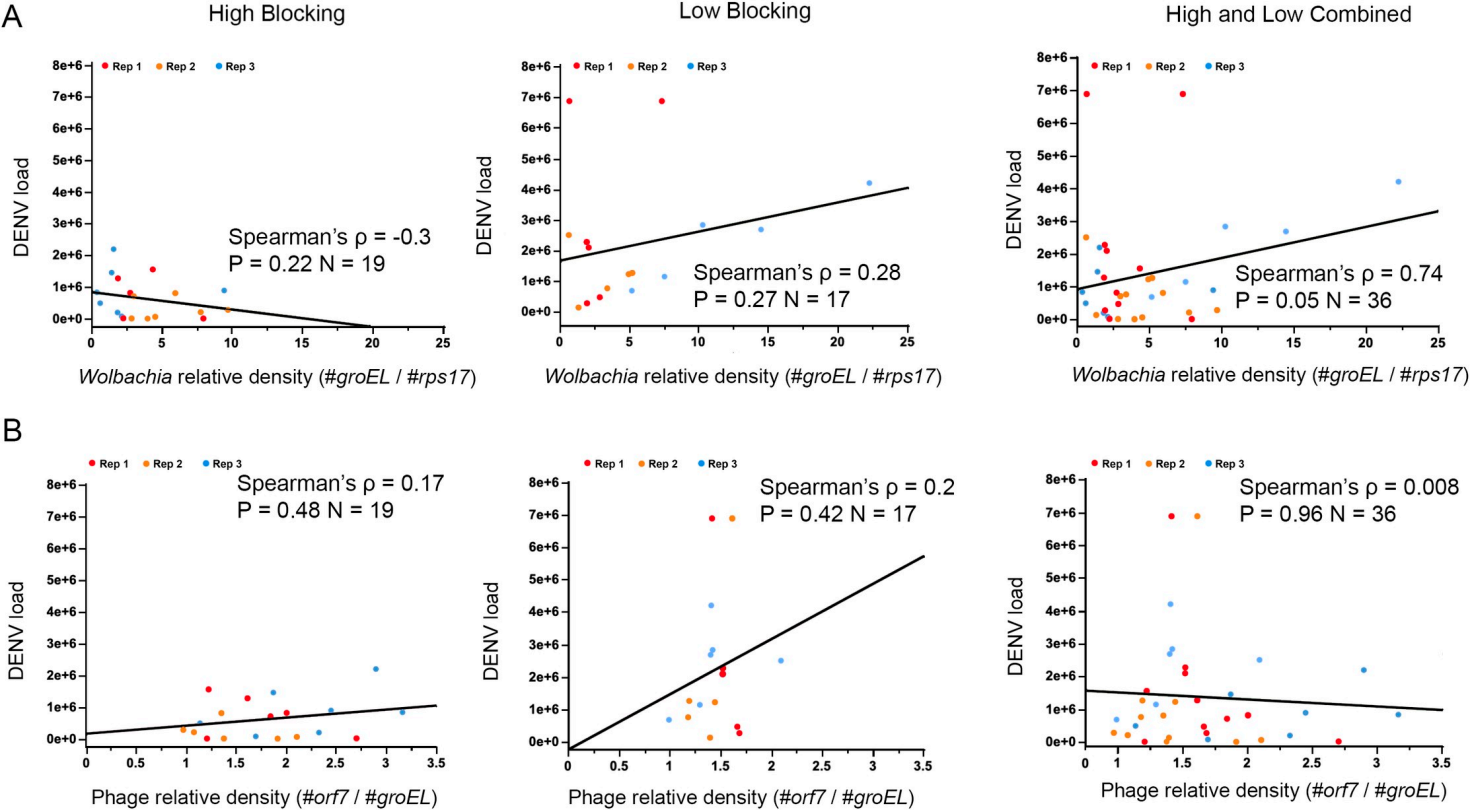
*Wolbachia* and phage WO relative densities do not associate with DENV load in *A*. *aegypti* populations after 4 generations of selection. Whole body (minus ovaries) DENV load, *Wolbachia* and phage WO relative densities in *w*Mel-infected *A*. *aegypti* mosquitoes from High and Low WMPB selected lines. Each dot represents a single mosquito. Red, orange and blue dots represent replicated populations 1, 2 and 3, respectively. Sample sizes are depicted within the figure for each group. Spearman’s rank correlation coefficient analyses were performed to describe the relationship between DENV load and (A) *Wolbachia* density (absolute copy number of *groEL* relative to the absolute copy number of *rps17*) and (B) phage density (absolute copy number of *orf7* relative to the absolute copy number of *groEL*) across evolved groups.

We then assessed if selection promoted changes in *Wolbachia* or phage WO relative densities that predicted differences in DENV load in the evolved lines. None of the selected lines displayed a significant correlation with DENV load for either *Wolbachia* densities (High: Spearman’s ρ = -0.3, P = 0.22/ Low: Spearman’s ρ = 0.28, P = 0.27) (Fig 3A), or phage WO densities (High: Spearman’s ρ = 0.17, P = 0.48 /Low: Spearman’s ρ = 0.2, P = 0.42) (Fig 3B). Unlike above, combining all selected lines did not result in a significant correlation with DENV load for either *Wolbachia* (Spearman’s ρ = 0.74, P = 0.05) (Fig 3A) nor phage WO (Spearman’s ρ = 0.008, P = 0.96) relative densities (Fig 3B).

### Low DENV blocking mosquitoes have reduced overall fitness and higher predicted deleterious mutation load

While we did not observe a linear relationship between phage/*Wolbachia* relative densities and DENV loads, there were lower phage densities in the Low lines alone. From the original study [40], the Low blocking line also exhibited a reduced population growth rate (*r*) compared to the High blocking line. Significant SNP variation from the Poolseq approach (100 individuals per line) differentiated the High and Low Blocking lines, which may explain both differences in blocking as well as fitness, with potential consequences for phage WO. As such, we explored if there were elevated patterns of mildly predicted deleterious alleles in the selected lines and across the different chromosomes. While there is no evidence of *A*. *aegypti* genes that affect phage WO density, stress in *Wolbachia* due to external influences, such as temperature exposure in the host, leads to activation of phage WO [43]. Similarly, physiological changes in *A*. *aegypti* cells brought on by deleterious genomic changes could theoretically have cascading impacts on phage WO activity, though cautiously there are other potential reasons for these associations as well.

**Fig 4:**
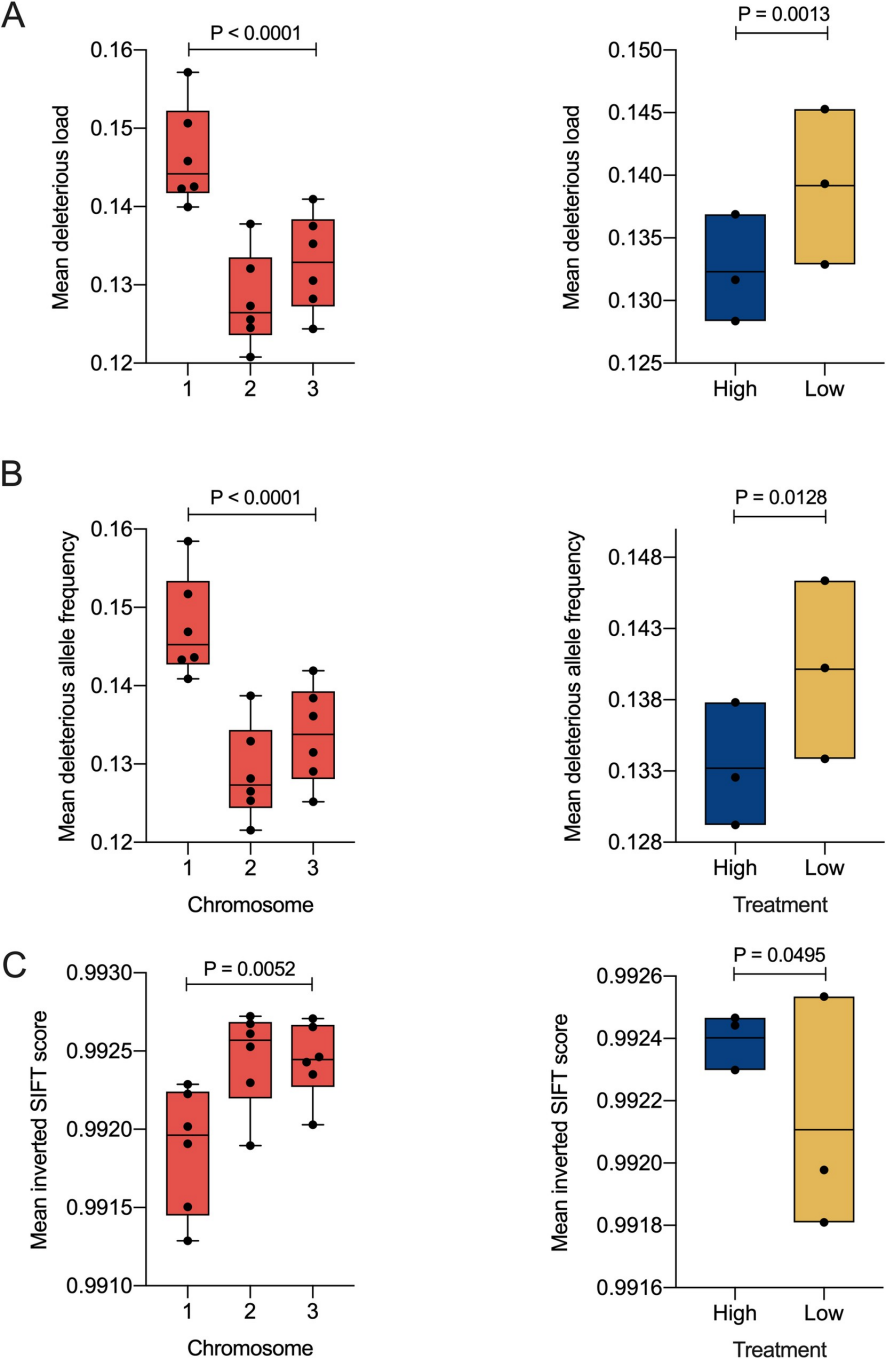
Low DENV blocker mosquitoes have a higher predicted deleterious mutation load than High DENV blockers; an effect primarily driven by higher allele frequency of potential deleterious SNPs on chromosome 1 in the Low blocking line. Boxplots represent screened *w*Mel-infected *A*. *aegypti* mosquitoes from WMPB selected lines for differences in (A) predicted mean deleterious load, (B) mean allele frequency of potential deleterious SNPs and (C) mean inverted SIFT score, as a proxy for accumulation of more severe SNPs across groups (High: dark blue—Low: yellow) and chromosomes (red). Significant comparisons expressed via P-values were determined via Type 2 and type 3 ANOVA and are listed in the results section of the main text. Each dot corresponds to a treatment group and its corresponded independent replicate line. N = 6 samples per chromosome and N = 3 samples per group for treatments in all graphs.

We quantified the potential deleterious effect of all protein coding SNPs and associated this with the population frequency to measure predicted deleterious load. Across all groups (High and Low blocking), we observed a significantly greater predicted mean deleterious load on chromosome 1 relative to chromosomes 2 and 3 (ANOVA, F = 20.62, d.f. = 2, P = 6.7x10^-5^) (Fig 4A–left panel). Additionally, Low DENV blocking lines on average displayed a significantly greater predicted mean deleterious mutation load than High DENV blockers (ANOVA, F = 8, d.f. = 1, P = 0.01) (Fig 4A–right panel). Further analysis revealed that this increase in the predicted mean deleterious load is primarily driven by higher mean allele frequency of predicted deleterious SNPs on chromosome 1 (ANOVA, F = 20.86, d.f. = 1, P = 6.3x10^-5^) (Fig 4B–left panel), and in the Low blocking line (ANOVA, F = 8.12, d.f. = 1, P = 0.013) (Fig 4B–right panel) rather than more severe predicted deleterious SNPs. This hypothesis is strengthened by evidence of a lower mean inverted SIFT score on chromosome 1 (ANOVA, F = 7.81, d.f. = 2, P = 0.005) (Fig 4C–left panel) and in Low blockers (ANOVA, F = 4.62, d.f. = 1, P = 0.049) (Fig 4C–right panel). From the original study, chromosome 1 is where the bulk of SNPs associating with differences between the High and Low blocking lines occurred [40].

### *cifA* and *cifB* expression pattern differs between somatic and reproductive tissues in *w*Mel-infected *A*. *aegypti* regardless of selection regime

**Fig 5:**
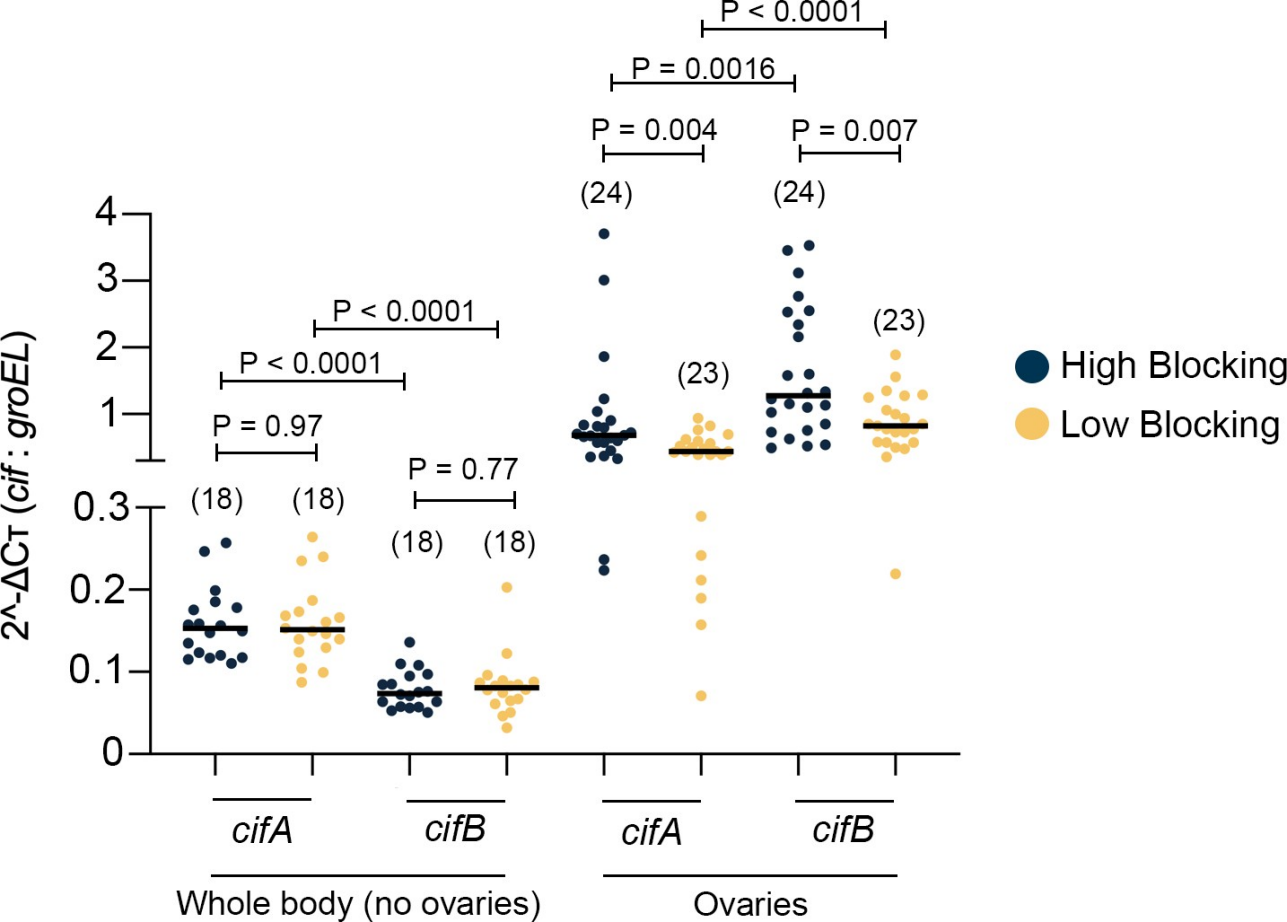
Expression of CI-inducing genes differs in reproductive but not somatic tissues of *w*Mel-infected *A*. *aegypti* from the 4^th^ generation of selection. Gene expression in whole body (minus ovaries) and ovaries of *w*Mel-infected *A*. *aegypti* mosquitoes selected for WMPB (High: dark blue—Low: yellow). Expression levels of *cifA* and *cifB* were normalized to *Wolbachia groEL* gene. Scatter plot depicts median levels of both genes for both the whole body and ovaries. Each dot represents a single mosquito. Sample sizes are depicted within the figure, in parentheses above each group, for the whole body minus ovaries and ovaries datasets. Significant comparisons expressed via P-values were determined via Kruskal-Wallis followed by post-hoc Dunn’s multiple comparison test.

Two key genes in the phage WO genome responsible for induction and rescue of CI are *cifA* and *cifB*. To test if changes in phage WO densities in the selected lines associate with phage-induced CI functions, and to examine a potential link between CI and WMPB, we performed whole-body gene expression analyses of the *cifA* and *cifB* genes in the selected lines. We observed significant differences in *cif* transcript levels in whole bodies (Kruskal-Wallis H = 43.7, d.f. = 3, P < 0.0001), with *cifA* expression significantly higher than *cifB* in all selected groups (High: Mann-Whitney U = 7, d.f. = 1; P < 0.0001 [2.1 fold]–Low: Mann-Whitney U = 21, d.f. = 1; P<0.0001 [1.9 fold]) (Fig 5). We also saw no differences in CI gene expression levels across selected lines for *cifA* (High vs. Low: Mann-Whitney U = 160.5, d.f. = 1; P = 0.97) or *cifB* (High vs. Low: Mann-Whitney U = 152.5, d.f. = 1; P = 0.77).

To explore if these findings extended to specific tissues, we analyzed ovaries because they are a key site for expression of *cifA*-mediated rescue of CI [20] and transmission of *Wolbachia* [44]. There were several significant differences (Kruskal-Wallis H = 33.8, d.f. = 3, P < 0.0001). *cifB* expression levels were higher than *cifA* in both selection groups (High: Mann-Whitney U = 138.5, d.f. = 1, P = 0.0016; [1.9 fold], and Low: Mann-Whitney U = 85.5, d.f. = 1, P < 0.0001; [1.9 fold]), and the expression levels for both genes were significantly higher in the High blocking group (Fig 5, *cif*A, Mann-Whitney U = 144, d.f. = 1, P< 0.004; *cifB*, Mann-Whitney U = 151, d.f. = 1, P = 0.007). Taken together across the two body samplings, these results demonstrate that the transcriptional regulation of the CI genes likely responded to selection for WMPB in a tissue-specific manner.

## Discussion

*Wolbachia* are currently at the forefront of vector control efforts to reduce transmission of arboviruses like dengue, Zika and Yellow fever to humans [12,13,45]. Successful field deployment of *Wolbachia*-infected mosquitoes in a population replacement strategy heavily relies on the long-term stability of both WMPB and CI. Previously, Ford *et al*., [40] demonstrated the potential for natural selection to maintain WMPB given a positive association with faster population growth. Here, aided by samples from this same experimental evolution study, we explored whether there is any evidence that temperate phage WO [26,46,47] play a role in *Wolbachia*-mediated DENV blocking.

We observed no evidence that phage WO relative densities in the somatic tissues predicted DENV load across selected lines, suggesting no direct link between phage load and WMPB. Our results did show both (i) a negative correlation between *Wolbachia* and phage WO relative densities when evolved populations were combined (Fig 2) and (ii) a positive correlation between *Wolbachia* and phage WO absolute abundance (Fig 1A and 1B), which is in line with previously-described dynamics in *N*. *vitripennis* parasitoid wasps [26,43]. These results support the “phage density model” in which activation of phage lytic particles corresponds to decreases in *Wolbachia* density [26,47].

As for *Wolbachia*, the lack of a significant correlation between its densities and pathogen blocking (Fig 3) is inconsistent with what has been described in *Drosophila* [48,49]. However, other experiments involving *A*. *aegypti* have also failed to detect a significant correlation between WMPB and bacterial density [15]. This lack of correlation is part of an increasing body of evidence suggesting that WMPB may arise as a collective result of many distinct mechanisms [37,38], not simply density.

Surprisingly, the selection regime drove differences in phage WO density between High and Low blocking lines, but not in *Wolbachia* densities. As our previous analysis of the selected lines [40] also did not detect significant SNPs in the *Wolbachia* genome, the change in phage WO relative density is not likely due to *Wolbachia*. We propose that the change in WO relative density between High and Low lines may result from SNPs in mosquito loci, resulting directly from selection from linkage with those under positive selection. Since our list of SNP candidates associated with changes in blocking come from Low vs High comparisons, it cannot be determined in which lineages the changes occurred. Any of the selected SNPs may also have pleiotropic effects on *Wolbachia* physiology and/or phage stability. Similarly, the selection regime may also have resulted in hitchhiking of mildly predicted deleterious alleles in linkage to key blocking SNPs. Co-authors of this study have previously reported that Low DENV blocking mosquitoes displayed a significantly lower average fitness when compared to High DENV blockers [40]. Our bioinformatic analyses here identified a higher predicted mean deleterious load in Low DENV blocking mosquitoes versus the High blocking line (Fig 4A). A significant number of genes on chromosome 1 [40], near the SNPs selected upon (Fig 4B), are involved in processes such as cell cycle, transcriptional pausing, oxidative stress and others [41]. The increase in genetic changes in chromosome 1 could have disrupted *Wolbachia’s* homeostasis, interfering with the balance between lytic and lysogenic cycles of the phage WO population. Phages are known to “sense” and respond to disturbances in the intracellular environment of the host where they reside [43]. Phage WO has numerous ankyrin repeat containing genes as well as other candidates, that appear to allow for direct protein-protein interaction with the host and that may provide a host sensing function [47,50–52]. For instance, The cell cycle has been directly associated with bacteriophage productivity in *Escherichia coli*, in which infections of larger cells late in the cell cycle resulted in a significant increase in phage numbers [53], with consequences for bacterial populations and host symbiosis.

The reduced phage density in the Low blocking line also corresponded, not surprisingly, to lower expression of both CI-inducing genes *cifA* and *cifB* in the ovaries, relative to the High blocking line. This is, to the best of our knowledge, the first time in which the expression of the CI genes has been quantitated in this tissue for mosquitoes. The observation of higher ovarian expression of *cifB* relative to *cifA* is surprising but plausible. Lindsey *et al*., observed higher *cifB* mRNA levels relative to *cifA* during the first two-thirds stages of embryogenesis, as well as during larval stages [54]. Work in *D*. *melanogaster* has shown that *cifA* expression levels are dramatically higher than *cifB* when analyzing 1, 3 or 7 days old adult individuals [21,54]. This observation also seems to be consistent with reports in 11–14 day old ovaries of *Drosophila pandora* infected with *w*PanCI and *w*PanMK [55], and with the observation here for whole body samples. Lindsey *et al*., detected the existence of an intergenic region between *cifA* and *cifB* in *w*Mel [54] that contains a hairpin termination signal that may impair the co-transcription of *cifA* and *cifB*. As such, the contrasting shift in expression pattern between these two genes could be attributed to yet unknown factors present in the ovaries that would regulate or interfere with such an intergenic regulatory region.

In conclusion, we propose that while WMPB and Phage WO load/CI-causing gene expression seem largely independent, there are potential scenarios involving pleiotropy or chromosomal linkage where selection on one trait may drive change in the other. Such relationships could lead to unforeseen consequences for *Wolbachia* release strategies. Future work should aim to understand the mechanistic nature of the blocking phenotype and how natural selection in the field may act upon the phenotype. Furthermore, the genomic region on chromosome 1 that is key to selected differences in blocking in our studies, should also be examined for potential roles in mosquito fitness and *Wolbachia*/phage WO homeostasis. Finally, without live male mosquitoes from the Ford *et al*. study we were unable to measure the strength of CI. Lower *cif* gene expression, however, would predict reduced CI in the Low lines, so future studies should assess the relationship between phenotypic shifts in blocking under selection and the expression of CI.

## Materials and methods

### Mosquitoes

We used 5–7 day old, mated and blood-fed female *A*. *aegypti* mosquito samples (whole body minus ovaries; ovaries collected separately) from a previous study [40]. Briefly, in that study, mosquitoes from an initial population (Ancestral) were exposed to an artificial selection regime for Low (L) and High (H) levels of *Wolbachia*-mediated dengue virus (serotype 3) blocking (WMPB), with a randomly passaged control population (R) that did not undergo a selection regime. Each treatment group (L, R and H), with the exception of the original Ancestral population, was represented by three independent replicate lines. Ford *et al* [40] performed selection based on total body load of DENV (RNA copies) as determined by quantitative reverse transcription polymerase chain reactions (qRT-PCR) carried out over four generations. Upon completion of selection, in addition to DENV blocking, *Wolbachia* density and a suite of mosquito fitness traits were measured in the treatment lines. The ideal control for comparison to the H and L blocking lines is the co-reared R blocking line, as it was replicated and will have captured any drift and off-target selection resulting from the breeding regime. However, our previous study [40] demonstrated that given an association between faster population growth and higher blocking, that laboratory rearing of the R line also co-selected for higher blocking. Since neither of the Ancestral or R lines are able to serve as true unselected controls, we have conservatively focused our comparisons between the evolved Low and High blocking lines, without attempting to interpret directionality of selection events.

### RNA and DNA extractions

Samples were divided into two categories: 1) whole body minus ovaries and 2) ovaries. Despite such division, the ovaries used here were not matched to the same mosquito whose whole body was analyzed, as only a small fraction of paired samples were collected in the original study, and those were depleted during the progression of the experiments in the original manuscript by Ford et al. Samples were homogenized in 200μL of TRIzol reagent (ThermoFisher Scientific) containing a 2.8mm DNase/RNase-free ceramic bead (VWR) and macerated using a bead ruptor elite (Omni International). Total RNA and DNA were extracted according to the manufacturer’s instructions. Nucleic acids were diluted in nuclease-free water and quantified using the NanoDrop 2000 spectrophotometer system (ThermoFisher Scientific). Each RNA sample was treated with DNase-I (Sigma) following the manufacturer’s instruction to ensure no genomic DNA contamination.

### *Wolbachia*, phage and mosquito gene quantification

*Wolbachia* density per mosquito host cell was obtained via quantitative polymerase chain reaction (qPCR) and quantifying the absolute copy numbers of the chaperonin *groEL* [see ref. [21] for the primer set] relative to the absolute copy numbers of *A*. *aegypti* ribosomal gene *rps17* [see ref [40] for the primer set] [26,43]. Unfortunately, due to lack of a complete genome sequences for both integrated prophage and active phage particles, there is no current way of precisely distinguishing between lysogenic and lytic particles for WOMelB phage. As such, phage density was quantified in a similar manner to *Wolbachia’s* relative quantification, using the absolute copy number of the phage capsid protein gene *orf7* (specific to WOMelB) relative to absolute copy numbers of *Wolbachia’s groEL* gene [43]. This quantification method potentially accounted, to a certain extent, to both integrated prophage sequences and putative lytic particles. Primers for *orf7* (113bp) [orf7Fw: 5’- TGGCGAGAAAGCAGTAGAAATA-3//orf7Rv: 5’- GGTCATTTGTAGTTTTTTCATTCATAG-3’] were designed using NCBI’s Primer-BLAST (www.ncbi.nlm.nih.gov/tools/primer-blast/) and both MFEPrimer 3.0 (mfeprimer.igenetech.com/) and IDT’s OligoAnalyzer tool (www.idtdna.com) for quality control. Samples for quantifying the aforementioned parameters were run in technical duplicates. Prior to use in experiments, all primer pairs used in this study were examined for both specificity and amplification efficiency [56]. Specificity analysis was performed by melt curve analysis, with primer pair displaying a single peak. Efficiency analysis was achieved by examining the amplification performance under a series of sample template dilutions.

Standards for all genes, *groEL*, *rps17* and *orf7* were obtained from IDT as gBlock double-stranded DNA gene fragments resuspended in UltraPure distilled water (Invitrogen) to an equivalent of 10^12^ copies of the genomic fragment. Standards were then aliquoted and frozen for one-time use. We used a total volume of 10μL per reaction, each containing: 5μL of 2x PerfeCTa SYBR Green SuperMix (Quantabio), 0.5μL of each forward and reverse primers (10μM), 2μL of nuclease-free water and 2uL of template DNA (100ng). Thermocycling conditions were as follows: initial denaturation at 95°C for 30 sec, and 45 cycles of 95°C for 3 sec and 60°C for 30 sec, followed by melt curve analysis using a LightCycler 480 Real-Time PCR system (Roche).

### Assessment of predicted deleterious load associated with SNPs in the *Ae*. *aegypti* genome

We have borrowed the concept of load from the field of population genetics, where load refers to the reduction in fitness of a population relative to population containing only individuals with perfect genotypes. More specifically, we have adapted the concept of mutation load to calculate what we refer to as the deleterious load [57]. The mutation load is measured as the negative selection coefficient, where larger values indicate that the mutation is more detrimental, multiplied by the allele frequency. Subsequently, the mutation load can increase either because the negative selection coefficient increases, or because the mutation increases in frequency within a population. To calculate the predicted deleterious load carried by each evolved replicate population, we substituted the negative selection coefficient used in mutation load with a measure of how deleterious a single nucleotide polymorphism (SNP) is and multiplied this by the population frequency of the SNP. The deleterious score of each SNP was acquired using the software SIFT (Sorting Intolerant From Tolerant) [58]. SIFT uses sequence homology across species to predict whether an amino acid or nucleotide substitution will affect protein function and hence, potentially alter the phenotype. The SIFT score ranges from zero to one and SNPs are by default categorized as deleterious if the score is less than 0.05.

Obtaining SIFT scores requires comparing SNPs of interest to a SIFT database. In order to build the SIFT database, we used the *A*. *aegypti* LVP_AGWG strain genome (*Aae*gL5) [59] and the UniProtKB/Swiss-Prot database [https://www.uniprot.org/] for the comparison to other species. Overall, of the 131,171,571 protein-coding positions in the *A*. *aegypti* genome, 115,419,545 (88%) received a SIFT score, with 64,101,512 of these (56%) receiving confident scores. Low confidence scores occur when the protein-coding sequences used for prediction are closely related, and many positions appear conserved, which can lead to false positives. In the proceeding analysis, we used all positions with SIFT scores irrespective of confidence. Once the SIFT database was built, SIFT scores for SNPs segregating in each evolved treatment/replicate were obtained via comparison of a Variant Call Format (VCF) file to the database.

In the previous analysis of this data, which identified cadherin as a candidate for resistance to DENV [40], SNPs were analyzed via a SYNC file [60,61] rather than a VCF file. Because a SYNC file cannot be readily converted to a VCF file, we had to recall SNPs. To call SNPs and produce a VCF file for this analysis, we used FreeBayes (Release 32) [62]. FreeBayes was run on each replicate with conservative settings to maximize the number of SNPs called (minimum alternate count of 1, minimum coverage of 5, maximum coverage of 200, and minimum base quality of 20). FreeBayes calls several different variant types including SNPs, multi-nucleotide polymorphisms (MNPs), insertions/deletions (INDELs), and complex haplotypes that can contain a combination of SNPs, MNPs, and INDELs. To decompose MNPs and complex haplotypes into SNPs, we used the vcfallelicprimitives and vcfbreakmulti functions from vcflib (github.com/vcflib/vcflib#vcflib). In order to produce the expected exponential distribution of alternate allele frequency in the Ancestral population, SNPs called by FreeBayes were further filtered to a minimum coverage of 20 and quality greater than 20 for the alternate call. Although each replicate’s sequencing coverage was similar, the number of SNPs called after quality filtering ranged from ~8M to ~4M. Reproduction of a similar Manhattan plot as Fig 4A in [40] confirmed that SNP frequencies called by FreeBayes were similar to those previously called.

To confirm that there was biological signal in the SIFT scores, we compared the frequency of SNPs classified as deleterious to those classified as tolerated in the Ancestral population. Under the probable assumption that the Ancestral population was at mutation-selection-drift balance, deleterious loci should be segregating at a lower allele frequency than tolerated loci. This was indeed the case (GLM: p = 9.2X10^-25^). To compare the predicted deleterious load of the High and Low DENV blocking treatments, load in each replicate was calculated using only those classified as potentially deleterious (SIFT score < = 0.05);

$$load=\frac{\sum_{i=1}^{n}(1-{SIFT}^{i})\times {frequency}^{i}}{n},$$

Where *SIFT^i^* is the SIFT score for a SNP, *frequency^i^* is the allele frequency of the SNP, and *n* is the total number of potential deleterious SNPs. Differences between selection treatments and chromosomes were then assessed via analysis of variance using the following linear model;

load=treatment+chromosome+treatment×chromosome+error.

Because the interaction was not significant (P = 0.94), the main effects of treatment and chromosome were assessed via type II sums of squares.

### Expression of cytoplasmic incompatibility factor genes cifA and cifB

Detection of the number of the CI-causing transcripts *cifA* (locus tag: WD_RS02835 –known as WD0631) and *cifB* (locus tag: WD_RS06940 –known as WD0632), was performed by utilizing previously published primer sets [54]. As a reference for transcription activity of *Wolbachia’s* core genome, we utilized the *Wolbachia groEL* gene. Machine-generated Cp values were used to calculate the relative expression of *cifA*:*cifB* using the **Δ**Ct (Livak) method. We set up qRT-PCRs using a LightCycler 480 Real-Time PCR system (Roche) and a total volume of 10μL per reaction, each consisting of 5μL of 2x qScript One-Step RT-qPCR SYBR Green Master Mix (Quantabio), 0.2μL of qScript Reverse Transcriptase enzyme (RT) (50x) (Quantabio), 0.2μL of each forward and reverse primers (10μM), 2.4μL of nuclease-free water and 2uL of template RNA (100ng). Thermocycling conditions were as follows: initial cDNA synthesis step at 50°C for 5min, RT deactivation/denaturation at 95°C for 2 min, and 45 cycles of 95°C for 3 sec and 60°C for 30 sec, followed by melt curve analysis.

### Statistical analyses

Normality for all *Wolbachia* and phage WO loads, as well as *cif* gene expression data were accessed using the Shapiro-Wilk test. Pairwise comparisons between non-normally distributed data were analyzed via Mann-Whitney’s U test, while multiple comparisons between non-normally distributed data were analyzed using Kruskal-Wallis followed by posthoc Dunn’s multiple comparison test with significance set at P < 0.05. Outliers were identified and removed prior to statistical comparisons, using 1% false discovery rate analyses, hence accounting for differences in sample size between datasets. Normally distributed data were compared with the Welch’s ANOVA followed by Dunnett’s T3 post hoc test with significance set at P < 0.05. Spearman’s rank correlation coefficient analysis was performed in order to describe the relationship between *Wolbachia* and phage density across distinct generations and selection groups. The DENV-3 x phage total abundance interaction was analyzed via Spearman’s rank correlation coefficient. All statistical analyses were performed using JMP Pro 15 (SAS) and Prism 8 (Graphpad).

## References

[1] Tolle MA. Mosquito-borne diseases. Curr Probl Pediatr Adolesc Health Care. 2009;39: 97–140. doi: 10.1016/j.cppeds.2009.01.001 19327647

[2] Messina JP, Brady OJ, Golding N, Kraemer MUG, Wint GRW, Ray SE, et al. The current and future global distribution and population at risk of dengue. Nat Microbiol. 2019;4: 1508–1515. doi: 10.1038/s41564-019-0476-8 31182801 PMC6784886

[3] Shepard DS, Undurraga EA, Halasa YA, Stanaway JD. The global economic burden of dengue: A systematic analysis. Lancet Infect Dis. 2016;3099: 1–7. doi: 10.1016/S1473-3099(16)00146-8 27091092

[4] Kraemer MUG, Reiner RC, Brady OJ, Messina JP, Gilbert M, Pigott DM, et al. Past and future spread of the arbovirus vectors *Aedes aegypti* and *Aedes albopictus*. Nat Microbiol. 2019;4: 854–863. doi: 10.1038/s41564-019-0376-y 30833735 PMC6522366

[5] da Silveira LTC, Tura B, Santos M. Systematic review of dengue vaccine efficacy. BMC Infect Dis. 2019;19: 750. doi: 10.1186/s12879-019-4369-5 31455279 PMC6712597

[6] Elsinga J, van der Veen HT, Gerstenbluth I, Burgerhof JGM, Dijkstra A, Grobusch MP, et al. Community participation in mosquito breeding site control: an interdisciplinary mixed methods study in Curaçao. Parasit Vectors. 2017;10: 434. doi: 10.1186/s13071-017-2371-6 28927437 PMC5606078

[7] Liu N. Insecticide resistance in mosquitoes: impact, mechanisms, and research directions. Annu Rev Entomol. 2015;60: 537–559. doi: 10.1146/annurev-ento-010814-020828 25564745

[8] Braga IA, Valle D. *Aedes Aegypti* insecticides, mechanisms of action and resistance. Epidemiol e Serviços Saúde. 2007;16: 279–293. doi: 10.5123/S1679-49742007000400006

[9] Wedell N, Price TAR, Lindholm AK. Gene drive: progress and prospects. Proc R Soc B Biol Sci. 2019;286: 20192709. doi: 10.1098/rspb.2019.2709 31847764 PMC6939923

[10] Macias VM, Ohm JR, Rasgon JL. Gene Drive for mosquito control: where did it come from and where are we headed? Int J Environ Res Public Health. 2017;14: 1006. doi: 10.3390/ijerph14091006 28869513 PMC5615543

[11] Huang Y-J, Higgs S, Vanlandingham D. Biological control strategies for mosquito vectors of arboviruses. Insects. 2017;8: 21. doi: 10.3390/insects8010021 28208639 PMC5371949

[12] Ryan PA, Turley AP, Wilson G, Hurst TP, Retzki K, Brown-Kenyon J, et al. Establishment of *w*Mel *Wolbachia* in *Aedes aegypti* mosquitoes and reduction of local dengue transmission in Cairns and surrounding locations in northern Queensland, Australia. Gates Open Res. 2020;3: 1547. doi: 10.12688/gatesopenres.13061.2 31667465 PMC6801363

[13] Nazni WA, Hoffmann AA, NoorAfizah A, Cheong YL, Mancini M V, Golding N, et al. Establishment of *Wolbachia* strain *w*AlbB in Malaysian Populations of *Aedes aegypti* for dengue control. Curr Biol. 2019;29: 4241–4248.e5. doi: 10.1016/j.cub.2019.11.007 31761702 PMC6926472

[14] Zug R, Hammerstein P. Still a host of hosts for *Wolbachia*: analysis of recent data suggests that 40% of terrestrial arthropod species are infected. PLOS One. 2012;7: e38544. doi: 10.1371/journal.pone.0038544 22685581 PMC3369835

[15] Dutra HLC, Rocha MN, Dias FBS, Mansur SB, Caragata EP, Moreira LA. *Wolbachia* blocks currently circulating Zika virus isolates in Brazilian *Aedes aegypti* mosquitoes. Cell Host Microbe. 2016;19: 771–774. doi: 10.1016/j.chom.2016.04.021 27156023 PMC4906366

[16] Teixeira L, Ferreira Á, Ashburner M. The bacterial symbiont *Wolbachia* induces resistance to RNA viral infections in *Drosophila melanogaster*. PLOS Biol. 2008;6: e1000002. doi: 10.1371/journal.pbio.1000002 19222304 PMC2605931

[17] Shropshire JD, Leigh B, Bordenstein SR. Symbiont-mediated cytoplasmic incompatibility: What have we learned in 50 years? Elife. 2020;9: 1–51. doi: 10.7554/eLife.61989 32975515 PMC7518888

[18] Caragata EP, Dutra HLC, Moreira LA. Exploiting intimate relationships: controlling mosquito-transmitted disease with *Wolbachia*. Trends Parasitol. 2016;32: 207–218. doi: 10.1016/j.pt.2015.10.011 26776329

[19] Zheng X, Zhang D, Li Y, Yang C, Wu Y, Liang X, et al. Incompatible and sterile insect techniques combined eliminate mosquitoes. Nature. 2019;572: 56–61. doi: 10.1038/s41586-019-1407-9 31316207

[20] Shropshire JD, On J, Layton EM, Zhou H, Bordenstein SR. One prophage WO gene rescues cytoplasmic incompatibility in *Drosophila melanogaster*. Proc Natl Acad Sci. 2018;115: 4987–4991. doi: 10.1073/pnas.1800650115 29686091 PMC5948995

[21] LePage DP, Metcalf JA, Bordenstein SR, On J, Perlmutter JI, Shropshire JD, et al. Prophage WO genes recapitulate and enhance *Wolbachia*-induced cytoplasmic incompatibility. Nature. 2017;543: 243–247. doi: 10.1038/nature21391 28241146 PMC5358093

[22] Metcalf JA, Jo M, Bordenstein SR, Jaenike J, Bordenstein SR. Recent genome reduction of *Wolbachia* in *Drosophila* recens targets phage WO and narrows candidates for reproductive parasitism. PeerJ. 2014;2: e529. doi: 10.7717/peerj.529 25165636 PMC4137656

[23] Shropshire JD, Leigh B, Bordenstein SR, Duplouy A, Riegler M, Brownlie JC, et al. Models and nomenclature for cytoplasmic incompatibility: caution over premature conclusions–a response to Beckmann et al. Trends Genet. 2019;35: 397–399. doi: 10.1016/j.tig.2019.03.004 31003827

[24] Beckmann JF, Bonneau M, Chen H, Hochstrasser M, Poinsot D, Merçot H, et al. Caution does not preclude predictive and testable models of cytoplasmic incompatibility: a reply to Shropshire et al. Trends Genet. 2019;35: 399–400. doi: 10.1016/j.tig.2019.03.002 30979535 PMC6525026

[25] Beckmann JF, Bonneau M, Chen H, Hochstrasser M, Poinsot D, Merçot H, et al. The toxin–antidote model of cytoplasmic incompatibility: genetics and evolutionary implications. Trends Genet. 2019;35: 175–185. doi: 10.1016/j.tig.2018.12.004 30685209 PMC6519454

[26] Bordenstein SR, Marshall ML, Fry AJ, Kim U, Wernegreen JJ. The tripartite associations between bacteriophage, *Wolbachia*, and arthropods. PLOS Pathog. 2006;2: e43. doi: 10.1371/journal.ppat.0020043 16710453 PMC1463016

[27] Masui S, Kuroiwa H, Sasaki T, Inui M, Kuroiwa T, Ishikawa H. Bacteriophage WO and virus-like particles in *Wolbachia*, an endosymbiont of arthropods. Biochem Biophys Res Commun. 2001;283: 1099–1104. doi: 10.1006/bbrc.2001.4906 11355885

[28] Wright JD, Sjostrand FS, Portaro JK, Barr AR. The ultrastructure of the rickettsia-like microorganism *Wolbachia pipientis* and Associated virus-like bodies in the mosquito *Culex pipiens*. J Ultrastruct Res. 1978;63: 79–85. doi: 10.1016/s0022-5320(78)80046-x 671578

[29] Bordenstein SR, Wernegreen JJ. Bacteriophage flux in endosymbionts (*Wolbachia*): Infection frequency, lateral transfer, and recombination rates. Mol Biol Evol. 2004;21: 1981–1991. doi: 10.1093/molbev/msh211 15254259

[30] Miao YH, Xiao JH, Huang DW. Distribution and evolution of the bacteriophage WO and Its antagonism with *Wolbachia*. Front Microbiol. 2020;11: 1–13. doi: 10.3389/fmicb.2020.00001 33281793 PMC7691483

[31] Wu M, Sun L V, Vamathevan J, Riegler M, Deboy R, Brownlie JC, et al. Phylogenomics of the reproductive parasite *Wolbachia pipientis w*Mel: a streamlined genome overrun by mobile genetic elements. PLOS Biol. 2004;2: e69. doi: 10.1371/journal.pbio.0020069 15024419 PMC368164

[32] Kent BN, Funkhouser LJ, Setia S, Bordenstein SR. Evolutionary genomics of a temperate bacteriophage in an obligate intracellular bacteria (*Wolbachia*). PLOS One. 2011;6: e24984. doi: 10.1371/journal.pone.0024984 21949820 PMC3173496

[33] Bordenstein SR, Reznikoff WS. Mobile DNA in obligate intracellular bacteria. Nat Rev Microbiol. 2005;3: 688–699. doi: 10.1038/nrmicro1233 16138097

[34] Chen H, Ronau JA, Beckmann JF, Hochstrasser M. A *Wolbachia* nuclease and its binding partner provide a distinct mechanism for cytoplasmic incompatibility. Proc Natl Acad Sci. 2019;116: 22314–22321. doi: 10.1073/pnas.1914571116 31615889 PMC6825299

[35] Shropshire JD, Bordenstein SR. Two-By-One model of cytoplasmic incompatibility: synthetic recapitulation by transgenic expression of cifA and cifB in *Drosophila*. PLOS Genet. 2019;15: e1008221. doi: 10.1371/journal.pgen.1008221 31242186 PMC6594578

[36] Amuzu HE, McGraw EA. *Wolbachia*-based dengue virus inhibition is not tissue-specific in *Aedes aegypti*. PLoS Negl Trop Dis. 2016;10: e0005145. doi: 10.1371/journal.pntd.0005145 27855218 PMC5113870

[37] Caragata EP, Rancès E, Hedges LM, Gofton AW, Johnson KN, O’Neill SL, et al. Dietary cholesterol modulates pathogen blocking by *Wolbachia*. PLOS Pathog. 2013;9: e1003459. doi: 10.1371/journal.ppat.1003459 23825950 PMC3694857

[38] Lindsey A, Bhattacharya T, Newton I, Hardy R. Conflict in the intracellular lives of endosymbionts and viruses: a mechanistic look at *Wolbachia*-mediated pathogen-blocking. Viruses. 2018;10: 141. doi: 10.3390/v10040141 29561780 PMC5923435

[39] Bhattacharya T, Newton ILG, Hardy RW. Viral RNA is a target for *Wolbachia*-mediated pathogen blocking. PLOS Pathog. 2020;16: e1008513. doi: 10.1371/journal.ppat.1008513 32555677 PMC7326284

[40] Ford SA, Allen SL, Ohm JR, Sigle LT, Sebastian A, Albert I, et al. Selection on *Aedes aegypti* alters *Wolbachia*-mediated dengue virus blocking and fitness. Nat Microbiol. 2019;4: 1832–1839. doi: 10.1038/s41564-019-0533-3 31451771 PMC6990461

[41] Ford SA, Albert I, Allen SL, Chenoweth SF, Jones M, Koh C, et al. Artificial selection finds new hypotheses for the mechanism of *Wolbachia*-mediated dengue blocking in mosquitoes. Front Microbiol. 2020;11. doi: 10.3389/fmicb.2020.01456 32733407 PMC7358395

[42] Bordenstein SR, Bordenstein SR. Eukaryotic association module in phage WO genomes from *Wolbachia*. Nat Commun. 2016;7: 13155. doi: 10.1038/ncomms13155 27727237 PMC5062602

[43] Bordenstein SR, Bordenstein SR. Temperature affects the tripartite interactions between bacteriophage WO, *Wolbachia*, and cytoplasmic incompatibility. PLOS One. 2011;6: e29106. doi: 10.1371/journal.pone.0029106 22194999 PMC3240643

[44] Frydman HM, Li JM, Robson DN, Wieschaus E. Somatic stem cell niche tropism in *Wolbachia*. Nature. 2006;441: 509–512. doi: 10.1038/nature04756 16724067

[45] O’Neill SL, Ryan PA, Turley AP, Wilson G, Retzki K, Iturbe-Ormaetxe I, et al. Scaled deployment of *Wolbachia* to protect the community from dengue and other Aedes transmitted arboviruses. Gates Open Res. 2019;2: 36. doi: 10.12688/gatesopenres.12844.3 30596205 PMC6305154

[46] Metcalf JA, Bordenstein SR. The complexity of virus systems: The case of endosymbionts. Curr Opin Microbiol. 2012;15: 546–552. doi: 10.1016/j.mib.2012.04.010 22609369 PMC3424318

[47] Kent BN, Bordenstein SR. Phage WO of *Wolbachia*: lambda of the endosymbiont world. Trends Microbiol. 2010;18: 173–181. doi: 10.1016/j.tim.2009.12.011 20083406 PMC2862486

[48] Osborne SE, Iturbe-Ormaetxe I, Brownlie JC, O’Neill SL, Johnson KN. Antiviral protection and the importance of *Wolbachia* density and tissue tropism in *Drosophila simulans*. Appl Environ Microbiol. 2012;78: 6922–6929. doi: 10.1128/AEM.01727-12 22843518 PMC3457512

[49] Martinez J, Ok S, Smith S, Snoeck K, Day JP, Jiggins FM. Should symbionts be nice or selfish? Antiviral effects of *Wolbachia* are costly but reproductive parasitism Is not. PLOS Pathog. 2015;11: e1005021. doi: 10.1371/journal.ppat.1005021 26132467 PMC4488530

[50] Klasson L, Kambris Z, Cook PE, Walker T, Sinkins SP. Horizontal gene transfer between *Wolbachia* and the mosquito *Aedes aegypti*. BMC Genomics. 2009;10: 33. doi: 10.1186/1471-2164-10-33 19154594 PMC2647948

[51] Masui S, Kamoda S, Sasaki T, Ishikawa H. Distribution and evolution of bacteriophage WO in *Wolbachia*, the endosymbiont causing sexual alterations in Arthropods. J Mol Evol. 2000;51: 491–497. doi: 10.1007/s002390010112 11080372

[52] Iturbe-Ormaetxe I, Burke GR, Riegler M, O’Neill SL. Distribution, expression, and motif variability of ankyrin domain genes in *Wolbachia pipientis*. J Bacteriol. 2005;187: 5136–5145. doi: 10.1128/JB.187.15.5136-5145.2005 16030207 PMC1196006

[53] Storms ZJ, Brown T, Cooper DG, Sauvageau D, Leask RL. Impact of the cell life-cycle on bacteriophage T4 infection. FEMS Microbiol Lett. 2014;353: 63–68. doi: 10.1111/1574-6968.12402 24822278

[54] Lindsey ARI, Rice DW, Bordenstein SR, Brooks AW, Bordenstein SR, Newton ILG. Evolutionary genetics of cytoplasmic incompatibility genes cifA and cifB in prophage WO of *Wolbachia*. Genome Biol Evol. 2018;10: 434–451. doi: 10.1093/gbe/evy012 29351633 PMC5793819

[55] Asselin AK, Villegas-Ospina S, Hoffmann AA, Brownlie JC, Johnson KN. Contrasting patterns of virus protection and functional incompatibility genes in two conspecific *Wolbachia* strains from *Drosophila pandora*. Appl Environ Microbiol. 2018;85: AEM.02290–18. doi: 10.1128/AEM.02290-18 30552191 PMC6384105

[56] Bustin SA, Benes V, Garson JA, Hellemans J, Huggett J, Kubista M, et al. The MIQE guidelines: minimum information for publication of quantitative real-time PCR experiments. Clin Chem. 2009;55: 611–22. doi: 10.1373/clinchem.2008.112797 19246619

[57] Whitlock M, Davis B. Genetic Load. eLS. 2011. doi: 10.1002/9780470015902.a0001787.pub2

[58] Ng PC, Henikoff S. SIFT: Predicting amino acid changes that affect protein function. Nucleic Acids Res. 2003;31: 3812–3814. doi: 10.1093/nar/gkg509 12824425 PMC168916

[59] Matthews BJ, Dudchenko O, Kingan SB, Koren S, Antoshechkin I, Crawford JE, et al. Improved reference genome of *Aedes aegypti* informs arbovirus vector control. Nature. 2018;563: 501–507. doi: 10.1038/s41586-018-0692-z 30429615 PMC6421076

[60] Kofler R, Pandey RV, Schlötterer C. PoPoolation2: identifying differentiation between populations using sequencing of pooled DNA samples (Pool-Seq). Bioinformatics. 2011;27: 3435–6. doi: 10.1093/bioinformatics/btr589 22025480 PMC3232374

[61] Kofler R, Orozco-terWengel P, de Maio N, Pandey RV, Nolte V, Futschik A, et al. Popoolation: A toolbox for population genetic analysis of next generation sequencing data from pooled individuals. PLOS One. 2011;6. doi: 10.1371/journal.pone.0015925 21253599 PMC3017084

[62] Garrison E, Marth G. Haplotype-based variant detection from short-read sequencing. arXiv. 2012; 1207–3907. Available: http://arxiv.org/abs/1207.3907

